# Are parental concerns for child TV viewing associated with child TV viewing and the home sedentary environment?

**DOI:** 10.1186/1479-5868-8-102

**Published:** 2011-09-27

**Authors:** Natalie Pearson, Jo Salmon, David Crawford, Karen Campbell, Anna Timperio

**Affiliations:** 1 School of Sport, Exercise & Health Sciences, Loughborough University, Epinal Way, Loughborough, Leicestershire, LE11 3TU, UK; 2 School of Exercise and Nutrition Sciences, Deakin University, 221 Burwood Highway, Burwood, Victoria, 3125, Australia

**Keywords:** Parents, Children, Television viewing, Sedentary behaviour, Home environment

## Abstract

**Background:**

Time spent watching television affects multiple aspects of child and adolescent health. Although a diverse range of factors have been found to be associated with young people's television viewing, parents and the home environment are particularly influential. However, little is known about whether parents, particularly those who are concerned about their child's television viewing habits, translate their concern into action by providing supportive home environments (e.g. rules restricting screen-time behaviours, limited access to screen-based media). The aim of this study was to examine associations between parental concerns for child television viewing and child television viewing and the home sedentary environment.

**Methods:**

Parents of children aged 5-6 years ('younger' children, n = 430) and 10-12 years ('older children', n = 640) reported usual duration of their child's television (TV) viewing, their concerns regarding the amount of time their child spends watching TV, and on aspects of the home environment. Regression analyses examined associations between parental concern and child TV viewing, and between parental concern and aspects of the home environment. Analyses were stratified by age group.

**Results:**

Children of concerned parents watched more TV than those whose parents were not concerned (B = 9.63, 95% CI = 1.58-17.68, p = 0.02 and B = 15.82, 95% CI = 8.85-22.80, p < 0.01, for younger and older children respectively). Parental concern was positively associated with younger children eating dinner in front of the television, and with parental restriction of sedentary behaviours and offering sedentary activities (i.e. TV viewing or computer use) as a reward for good behaviour among older and young children. Furthermore, parents of older children who were concerned had fewer televisions in the home and a lower count of sedentary equipment in the home.

**Conclusions:**

Children of concerned parents watched more TV than those whose parents who were not concerned. Parents appear to recognise excessive television viewing in their children and these parents appear to engage in conflicting parental approaches despite these concerns. Interventions targeting concerned parents may be an innovative way of reaching children most in need of strategies to reduce their television viewing and harnessing this parental concern may offer considerable opportunity to change the family and home environment.

## Introduction

Television viewing is the most prevalent sedentary behaviour for young people in industrialised countries, and for many the most prevalent leisure time activity [1,2]. Evidence suggests that many young people far exceed the recommended two hours per day of total screen time in front of the television alone [3-7]. Time spent watching television affects multiple aspects of child and adolescent health [8]. High levels of television viewing are associated with negative effects on sleep, attention, interpersonal relationships [9] aggression, sexual behavior, substance use, disordered eating, academic difficulties [10], unhealthy eating and excess weight [11-15]. Furthermore, children who are high television viewers tend to remain high television viewers, relative to others over time [16], and high levels of television viewing in childhood are associated with health risk factors (e.g. overweight, poor cardiorespiratory fitness) in adulthood [11], independent of adult levels of television viewing [17]. The development of effective strategies and interventions to prevent excessive television viewing among young people requires a detailed understanding of the determinants of this behaviour.

Although a diverse range of factors have been found to be associated with young people's television viewing [18,19], the home environment is particularly influential. Children's health behaviours, including television viewing, evolve within the context of the home and family environment, and are influenced by parents' beliefs, attitudes and behaviours [20]. Previous research has identified numerous pathways by which parents may shape sedentary behaviour patterns, including parental modelling, rules around sedentary behaviour, availability and accessibility of screen-based equipment in the home, and parental attitudes and beliefs. For example, recent research has shown that family television viewing, an opportunity for parental modelling, is positively associated with children's television viewing [18,21] and that parental rules that restrict screen time are negatively associated with television viewing among children and adolescents [18,21,22]. Research has also shown that many young people have television sets in their bedrooms [4], which may be positively associated with television viewing time, particularly among older children and adolescents [18,19,23,24]. Furthermore, parents with low levels of self-efficacy to influence a child's physical activity and to control child's screen time are more likely to have children who exceed screen-time recommendations [25-27].

While it appears that parents play a significant role in their child's television viewing habits, little is known about whether parents, particularly those who are concerned about their child's television viewing habits, translate their concern into action by providing supportive home environments (e.g. rules restricting screen-time behaviours, limited access to screen-based media). Ecological systems theory suggests that parenting practices and behaviours are influenced directly by forces emanating from within the individual parent (i.e. their attitudes, concerns, personality etc.) [28,29]. Previous research has shown that parental concern for healthy eating is associated with a positive home food environment (e.g. availability of fruit and vegetables) [30]. However, parental concerns for adolescent weight have been shown to be associated with less supportive feeding practices [31], parental concern about their child's physical activity levels have been shown to be associated with a less supportive home environment for physical activity [32], and parental concern for television viewing has been associated with an increased likelihood of children eating in front of the television [33]. Such findings suggest that concerned parents may be aware of a problem (e.g. their child watches a lot of television), and that the impetus for parents to enact on their child's TV viewing may be operationalised in terms of concern levels. These levels of concern may be based on a personal belief about TV viewing and may also be stimulated by their child's actual viewing levels. Thus, parents who are 'concerned' about their child's physical activity and television viewing may be important and receptive targets of interventions aiming to support changes to children's behaviour. However, little is known about the home environment within families of parents who are concerned about their child's television viewing. Identifying such parents and assessing whether their concerns are reflected in supportive home environments may provide useful avenues for the development of future targeted interventions.

The current study fills a gap in the existing literature by exploring (i) associations between parental concerns about child television viewing and actual child television viewing, and (ii) associations between parental concern and the home sedentary environment among 5-6 and 10-12 year-old children.

## Methods

### Participants

Data were drawn from the Health Eating and Play study. In 2002/03, 13 state or Catholic elementary schools in metropolitan Melbourne, Australia, with enrolments greater than 200 students, were randomly selected from postcodes from the highest, middle and lowest quintiles of area-level socioeconomic disadvantage [34]. Twenty-four schools (nine in high, seven in middle, and eight in low socioeconomic status (SES) areas) agreed to participate (62% response rate from schools). All families of children in their first year of elementary/primary school (5-6 years; younger children) at all 24 schools and all families of children in grades 5-6 (10-12 years; older children) at 17 of the 24 schools were invited to take part.

This study was approved by the Deakin University Human Research Ethics Committee, the Victorian Department of Education and Training and the Catholic Education Office. All eligible children received a package to take home for a parent or guardian. Under existing ethical guidelines, it was necessary to seek active written consent from parents for each child's participation, and no information could be accessed regarding characteristics of non-respondents. Written parental consent was received for 1562 children (42% response). No area-level socioeconomic gradient was noted in response rates (41% response at high, 39% middle, and 48% in low SES areas). Due to incomplete data for one or more of the variables of interest, 434 children were excluded from analyses for this paper.

## Measures

### Parent questionnaire

All data were provided by the child's main caregiver, who completed a questionnaire at home. Respondents reported on their own behalf and, where applicable, on behalf of their partner. Parents reported their age, gender, language usually spoken at home (categorised as English speaking or non-English speaking), marital status, and highest level of education attained. Based on reported gender of the respondent and co-caregiver, maternal (mother or female caregiver) education was derived. For the present study, maternal education was collapsed into three categories: some secondary school or less (low maternal education); completed secondary school, tertiary certificate, or apprenticeship (medium maternal education); and university/tertiary qualification (high maternal education). In addition, parents reported the gender and the date of birth of their child.

All questionnaire items underwent test-retest reliability testing as part of this study. A random subsample of 176 study parents completed the original questionnaire a second time two weeks after they had completed the initial questionnaire. Intra-class correlations (ICCs) and percent agreement were used to assess test-retest reliability. All items used in this study have acceptable reliability (ICC = 0.43-0.99) [32,35].

### Parental concern

To assess parental concerns, respondents were asked one question: 'How concerned are you that your child watches too much television?' Response options were given on a four-point Likert scale, ranging from (1) 'not concerned' to (4) 'very concerned'.

### Home sedentary environment

Respondents were asked one question regarding their own values about TV viewing: 'How much do you personally care about how much time you spend watching TV?' Response options were given on a four-point Likert scale: (1) 'not at all' (2) 'a little' (3) 'quite a bit' (4) 'very much'.

Respondents were asked five questions regarding modeling of sedentary behaviours and two questions regarding their child's eating while watching TV (see Table 1). Response options were given on a 5-point Likert scale: (1) 'never or rarely' (2) 'less than once a week' (3) 'once a week' (4) 'about 2-3 times a week' (5) 'about 4-6 times a week' and (6) 'everyday'.

**Table 1:** Description of the home sedentary environment of younger and older children

	Young children (n = 450)	Older children (n = 678)	p-value
**Home environment (mean (SD))**			

**Parent values **(range: 1-4)			

Parent cares about the amount of time they themselves spend watching TV	2.30 (0.58)	2.28 (0.59)	0.62

**Parent modelling **(range 1-6)			

Parent watched TV, videos or DVD's with the child	3.27 (1.13)	3.65 (1.36)	**< 0.001**

Parent used computer or internet with the child	2.27 (1.18)	2.30 (1.19)	0.71

Parent played electronic games with the child	1.60 (1.01)	1.50 (0.94)	0.11

Parent ate dinner in front of TV with the child	2.19 (1.62)	2.44 (1.70)	**0.01**

Parent ate snacks with child while watching TV	2.07 (1.23)	2.20 (1.27)	0.07

**Child eating while watching TV **(range 1-6)			

Child ate dinner in front of TV	2.43 (1.76)	2.66 (1.73)	**0.04**

Child ate snacks while watching TV	3.40 (1.57)	3.52 (1.58)	0.19

**Parenting practices**			

Parents are restrictive about sedentary behaviours (range: 6-30)	23.4 (5.80)	23.1 (5.77)	0.37

Parents offer sedentary behaviour as a reward (range: 2-10)	4.37 (2.60)	3.85 (2.43)	**0.001**

**Home sedentary environment**			

Three or more televisions in home (% yes)	38.5	55.3	**< 0.001**

Television in child's bedroom (% yes)	14.0	28.3	**< 0.001**

Computer or e-game console in child's bedroom (% yes)	14.5	29.1	**< 0.001**

Overall count of sedentary equipment (range: 1-10)	5.5 (1.56)	6.38 (1.53)	**< 0.001**

Pearson's X^2 ^test of significance for categorical variables (three of more televisions in home, television in child's bedroom and computer or e-game console in child's bedroom); Independent t-tests for continuous variables

Respondents were asked six questions regarding their sedentary-related restrictive parenting practices and two regarding their use of sedentary behaviour as a reward, adapted from the Child Feeding Questionnaire (CFQ) [36]. Items related to restrictive parenting practices included: (i) 'I have to be sure that my child does not watch too much TV', (ii) 'I have to be sure that my child does not spend too much time on the computer/internet', (iii) 'I have to be sure that my child does not spend too much time playing electronic games', (iv) 'I will switch off the TV if I think my child is watching too much', (v) 'I restrict how much time my child spends watching TV', (vi) 'I restrict how much time my child spends using the computer and playing electronic games'. Items related to using sedentary behaviour as a reward included: (i) 'I let my child watch TV in exchange for good behaviour', (ii) 'I let my child use the computer/internet or play electronic games in exchange for good behaviour'. Response options were provided on a 5-point Likert scale (scoring in parentheses): (1) 'Disagree' (2) Slightly disagree' (3) 'Neutral' (4) 'Slightly agree' (5) 'Agree'. The score of items related to restrictive parenting practices and use of sedentary behaviour as a reward, respectively, were summed and internal reliability of the scales were high (Cronbach's alpha: 0.81-0.83).

To assess opportunities for sedentary behaviour in the home, respondents were asked to report the presence of televisions and other electronic entertainment devices (e.g. DVD player, computer, pay TV) in the home. The number of checked items was summed to create a sedentary access score (range 1-10). Respondents were also asked how many televisions were in the family home (dichotomized as three or more televisions in the home/fewer than 3 televisions), and whether the child had a television and/or computer/electronic games console in their bedroom (dichotomized as yes/no).

### Child television viewing

Respondents reported the amount of time their child spends watching television (including commercial, non-commercial, cable/pay TV, videos, and DVDs) on a usual school day and usual weekend day (scale ranging from 0 to 6 or more hours, in half hour segments). School day estimates were multiplied by 5, and weekend day estimates were multiplied by 2; the totals were summed and divided by 7 to generate average viewing time (minutes per day).

### Child weight status

Height and weight without shoes were measured in private, at the child's school, by trained researchers using digital scales and a portable stadiometer. Body mass index (BMI = weight [kg]/height [m^2 ^]) was calculated and children were dichotomised into two groups 'not overweight' and 'overweight/obese' based on internationally accepted age- and sex-specific cut-off points [37].

### Statistical analyses

All analyses were conducted using Stata 11 (Stata Corp, College Station TX, 2003). Descriptive statistics were used to summarise the demographic and TV viewing characteristics of the sample. Pearson's X^2 ^tests were used to examine differences in the home sedentary environment according to child age group. Linear regression analyses were conducted to examine the association between parental concerns and child TV viewing. Separate, linear regression models were conducted to examine the association between parental concern and each of the home sedentary environment variables. All regression models were adjusted for child gender, weight status, television viewing (mins/day) and maternal education, and accounted for potential clustering by school (unit of recruitment) using the 'cluster' command.

## Results

Characteristics of the 1128 children in the sample are presented in Table 2. In both age groups, the sample was distributed across maternal education categories, providing a socio-economically diverse sample. Mean daily television viewing for the total sample exceeded 3 hours and was higher in older children.

**Table 2:** Characteristics of participants

	Total (n = 1128)	Younger children (n = 450)	Older children (n = 678)
**Sex (% boys)**	49	52	47

**Maternal education**			

Low	22	22	22

Medium	40	39	40

High	38	39	38

**TV viewing (mins/day)**	186.20 (93.07)	164.37 (87.20)	200.74 (94.08)***

Pearson's X^2 ^tests of significance, Independent t-tests for TV viewing (continuous variable)

*p < 0.05, **p < 0.01, ***p < 0.001

Parents of older children reported higher levels of concern than parents of younger children (mean(SD) = 2.04 (0.97) vs. mean(SD) = 1.85(0.98), p = 0.002). After adjusting for child gender, weight status, maternal education, linear regression analyses showed that parental concern for child TV viewing was significantly associated with child TV viewing (B = 9.63, 95% CI = 1.58-17.68, p = 0.02 and B = 15.82, 95% CI = 8.85-22.80, p < 0.001 for younger and older children respectively).

There were many differences in the home sedentary environment according to child age group (see Table 1). Parents of older children reported watching TV, videos or DVD's together with their child, and eating dinner in front of the TV together with their child more often than parents of younger children. Parents of older children reported that their child ate dinner in front of the TV more often than parents of younger children. Parents of younger children reported offering sedentary behaviour as a reward more often than parents of older children. A higher percentage of parents of older children reported that they had three or more TV's in the home, a TV in the child's bedroom, a computer or e-game console in the child's bedroom and a higher overall count of sedentary equipment in the home.

Tables 3 and 4 show the results of linear regression models for the associations between parental concerns and the home sedentary environment among younger and older children. After adjusting for child gender, weight status, television viewing (mins/day) and maternal education, regression analyses showed that parental concerns were associated with four factors in the home environment among younger children (Table 3). Parental concern was positively associated with the frequency of their child eating dinner in front of the TV, and with the use of restrictive parenting practices and the use of sedentary behaviour as a reward. Parental concern was also associated with having fewer televisions in the home.

**Table 3:** Associations between parental concerns and the home environment of younger children (n = 450).

	Parental concern		
	**Regression coefficient (SE)**	**95% CI**	**p**

**Home sedentary environment**			

**Parent values**			

Parent cares about the amount of time they themselves spend watching TV (n, % cares a lot)	0.01 (0.03)	-0.06-0.08	0.87

**Parent modelling**			

Parent watched TV, videos or DVD's with the child	0.01 (0.06)	-0.11-0.13	0.86

Parent used computer or internet with the child	-0.04 (0.05)	-0.15-0.06	0.41

Parent played electronic games with the child	0.01 (0.04)	-0.07-0.09	0.89

Parent ate dinner in front of TV with the child	0.01 (0.06)	-0.11-0.13	0.87

Parent ate snacks with child while watching TV	0.03 (0.07)	-0.10-0.17	0.61

**Child eating while watching TV**			

Child ate dinner in front of TV	**0.18 (0.09)**	**-0.002-0.35**	**0.05**

Child ate snacks while watching TV	0.11 (0.08)	-0.05-0.27	0.17

**Parenting practices**			

Parents are restrictive about sedentary behaviours	**1.97 (0.27)**	**1.40-2.53**	**< 0.001**

Parents offer sedentary behaviour as a reward for good behaviour	**0.49 (0.17)**	**0.13-0.85**	**0.01**

**Home sedentary environment**			

Three or more televisions in home	**-0.05 (0.03)**	**-0.11-0.0001**	**0.05**

Television in child's bedroom	-0.05 (0.03)	-0.12-0.01	0.09

Computer or e-game console in child's bedroom	-0.03 (0.02)	-0.07-0.01	0.15

Overall count of sedentary equipment	-0.06 (0.06)	-0.19-0.07	0.33

Linear regression analyses adjusted for child gender, weight status, TV viewing (mins/day), maternal education and accounted for potential clustering by school (unit of recruitment) using the 'cluster' command. **Bold **text indicates significant associations.

**Table 4:** Associations between parental concerns and home environment among older children (n = 678).

	Parental concern		
	**Regression coefficient (SE)**	**95% CI**	**p**

**Home sedentary environment**			

**Parent values**			

Parent cares about the amount of time they themselves spend watching TV (n, % cares a lot)	0.01 (0.03)	-0.04-0.07)	0.63

**Parent modelling**			

Parent watched TV, videos or DVD's with the child	-0.06 (0.06)	-0.18-0.07	0.36

Parent used computer or internet with the child	-0.02 (0.04)	-0.11-0.08	0.72

Parent played electronic games with the child	0.03 (0.05)	-0.07-0.14	0.50

Parent ate dinner in front of TV with the child	0.04 (0.06)	-0.08-0.17	0.48

Parent ate snacks with child while watching TV	0.03 (0.05)	-0.07-0.13	0.49

**Child eating while watching TV**			

Child ate dinner in front of TV	0.09 (0.06)	-0.04-0.23	0.17

Child ate snacks while watching TV	0.06 (0.05)	-0.05-0.18	0.27

**Parenting practices**			

Parents are restrictive about sedentary behaviours	**2.29 (0.23)**	**1.78-2.79**	**< 0.001**

Parents offer sedentary behaviour as a reward for good behaviour	**0.67 (0.11)**	**0.43-0.91**	**< 0.001**

**Home sedentary environment**			

Three or more televisions in home	**-0.05 (0.01)**	**-0.08- -0.02**	**0.002**

Television in child's bedroom	0.03 (0.03)	-0.03-0.09	0.27

Computer or e-game console in child's bedroom	-0.004 (0.01)	-0.03-0.02	0.68

Overall count of sedentary equipment	**-0.16 (0.06)**	**-0.28- -0.04**	**0.01**

Linear regression analyses adjusted for child gender, weight status, TV viewing (mins/day), maternal education and accounted for potential clustering by school (unit of recruitment) using the 'cluster' command. **Bold **text indicates significant associations.

After adjusting for child gender, weight status, television viewing (mins/day) and maternal education, regression analyses showed that parental concerns were associated with four factors of the home environment among older children (Table 4). Parental concern was positively associated with the use of restrictive parenting practices and the use of sedentary behaviour as a reward. Parental concern was also associated with having fewer televisions in the home and a lower count of sedentary equipment in the home.

## Discussion

This study examined whether parental concern for child television viewing was associated with this behaviour, and whether parental concerns for child television viewing were associated with the home sedentary environment. This study found that parental concern was positively associated with television viewing among younger and older children. In addition, despite their concerns, certain aspects of the home environment were not as favourable among concerned parents as those of parents who were not concerned. These findings suggest that parents who are concerned about their child's TV viewing have reason to be and that they may not be aware of the role of certain parenting practices on their child's television viewing. Thus, family-based interventions that provide education, support or encouragement to concerned parents to enact changes to the family environment may be an important approach to reducing excessive television viewing in children. In addition, targeting parents who are concerned about their child's television viewing may be an effective strategy for reaching children who are most in need and parents who express concern may be particularly receptive to interventions.

Although on average children of concerned and unconcerned parents watched more television than is currently recommended, the present study suggests that parents of both younger and older children are able to recognise excessive television viewing in their child since parental concern distinguished those that watched the most from those that watched the least television. This is consistent with previous findings from the HEAPs study that showed that parents who were concerned about their child's physical activity had less active children as measured by accelerometry [32]. The higher prevalence of concern among parents of older children in the present study reflects the higher amount of television viewing among these children compared to the younger children in the study.

Despite concerns about their child's television viewing, parental concern was positively associated with the frequency of their child eating dinner in front of the TV among parents of younger children. Children are exposed to numerous advertisements when watching television and these are known to influence the type of food desired, requested and consumed [38]. Furthermore, it is posited that eating while watching television may stimulate overconsumption of food and increased energy intake [39]. Early childhood research suggests that young people may associate television viewing with eating from a young age, if for example, parents place their children in front of the television with a snack or a meal while they do other household chores [40]. Research has shown that turning off the television during dinner is related to higher diet quality among parents [41] and children [42,43], and with lower levels of television viewing [44,45]. Strategies that encourage parents to eat meals together with their child without the television on are warranted, particularly among concerned parents of younger children.

Parental concern was positively associated with the use of restrictive parenting practices related to television access among parents of younger and older children. Although cross-sectional, parental restriction of viewing may be a direct response to a child's excess viewing. In previous studies, restrictions and rules around sedentary behaviours, such as television viewing, have been associated with lower levels of television viewing [21,46]. In contrast, parental concern was positively associated with offering sedentary behaviours as a reward to their child for good behaviour among parents of younger and older children. Offering sedentary behaviour as a reward seems at odds to the use of restrictive practices and may send mixed messages to the child and inadvertently increase television viewing time. Consistent with previous research [33], our findings suggest that parents have come to depend on television despite their misgivings about it. Interventions that provide parents with strategies to enforce rules and restrictions and to reduce their reliance on sedentary behaviour as a reward are needed. Parents, for example, could offer active rewards, such as a park visit.

Again, in contrast to the use of TV as a reward for good behaviour, concerned parents of younger and older children reported having fewer TVs in the home and less sedentary equipment (among older children only). Whether parents have reduced the availability of TVs and sedentary equipment in the home in response to their concerns, or whether concerned parents have a more set attitude towards TV viewing irrespective of their child's viewing habits is unclear. However, given the positive association between parental concern and the child's TV viewing time, the latter scenario is a less likely explanation.

Strengths of this study include the large sample of children which allowed stratification by age group, the socioeconomic diversity of the sample, and the comprehensive examination of the home sedentary environment. However, there were some limitations, including the modest response rate (although this was similar to that achieved in other health surveys), and reliance on parental report of television viewing, which may be less accurate among parents of older children. Furthermore, it may be that children whose parents are not concerned may be less aware of their child's television viewing patterns, particularly if their child watches television on their own or with other children. Future studies should include TV diaries to overcome some of these limitations and to confirm the finding that children whose parents are concerned about their television viewing watch more television than children whose parents report not being concerned.

## Conclusion

Parents appear to recognise excessive television viewing in their children and these parents appear to engage in conflicting parental approaches despite these concerns. Parents who are concerned about their child's television viewing behaviours could benefit from messages focusing on turning the television off during meals, and discouraging eating while watching television. Furthermore, strategies to encourage parents to enforce rules regarding television and other screen-based media and to reduce their reliance on the use of sedentary behaviour as a reward could affect television viewing patterns among children whose parents are concerned. Interventions targeting concerned parents may be an innovative way of reaching children most in need of strategies to reduce their television viewing and harnessing this parental concern may offer considerable opportunity to change the family and home environment.

## Competing interests

The authors declare that they have no competing interests.

## Authors' contributions

NP and AT conceived the manuscript. NP analyzed the data and drafted the manuscript. AT, DC and JS designed the Health Eating and Play study (HEAPs) project, and all authors provided critical feedback on drafts and read and approved the final manuscript.
